# Pathogenic Fungal Species Associated with Digestive System of *Periplaneta americana* (Blattaria: Blattidae) Trapped from Residential Dwellings in Ahvaz City, Southwestern Iran

**Published:** 2018-03-18

**Authors:** Hamid Kassiri, Majid Zarrin, Rahele Veys-Behbahani

**Affiliations:** 1 Medical Entomology and Vector Control Department, Health Faculty, Ahvaz Jundishapur University of Medical Sciences, Ahvaz, Iran; 2 Medical Mycology Department, Medicine Faculty, Ahvaz Jundishapur University of Medical Sciences, Ahvaz, Iran; 3 Student Research Committee, Ahvaz Jundishapur University of Medical Sciences, Ahvaz, Iran

**Keywords:** *Periplaneta americana*, American cockroach, Residential environments, Fungal flora, Isolation

## Abstract

**Background:**

Cockroaches are the most prevalent domestic pests of a worldwide distribution. They were recognized as possible vectors of pathogenic bacteria, viruses, fungi and parasites in residential dwellings and hospital environments. The present study isolated and identified yeasts and filamentous fungi from digestive tract of American cockroaches, collected from three different residential regions of Iran.

**Methods:**

Seventy cockroaches were sampled using direct collection (hand catch), vacuum cleaner and sticky traps in Ahvaz, Iran in 2009–2010. Their medically important fungal microorganisms were isolated from digestive tract using standard mycological methods. Filamentous fungi were identified by macroscopic and microscopic examination. Yeasts were identified by API ID32C-32100 kit.

**Results:**

A high percentage of cockroaches (88.6%) were detected to carry fungi of medical importance. Overall, 23 fungi species/genera were isolated from the American cockroaches' alimentary tract. The fungi isolated from cockroaches, from the residential regions were species of *Aspergillus*, *Rhizopus*, *Penicillium*, *Mucorales*, *Alternaria*, *Cladosporium*, *Mycelia*, *Chrysosporium*, *Candida*, *Rhodotorula*, *Zygosaccharomyces*, and *Debaryomyces*. *Candida* spp. (41.4%), *Aspergillus* spp. (37.1%) and *Rhodotorula* spp (27.1%) were the most common fungi recovered on cockroaches. *Candida albicans* and *Candida glabrata* were the commonest species of the genus *Candida*. In addition, *Aspergillus niger* and *A. flavus* were the most frequent species of the genus *Aspergillus*.

**Conclusion:**

American cockroaches may carry pathogenic fungi in the urban areas of Ahvaz.

## Introduction

Cockroaches are distinguished by the subsequent characteristics: wings and tarsus, reproductive organs, head frontal, shape and the number of spines on the femora and color. They comprise five families of Ectobiidae (Blattellidae), Blaberidae, Cryptocercidae, Blattidae and Corydiidae (Polyphagidae) (1). Cockroaches can be detected in a broad range of surroundings all over the globe, particularly in tropical and subtropical areas. More than 4500 species are reported from different parts of the world. They are one of the most generally prominent household pests and about 30 species are associated with human dwellings (1, 2). House cockroaches such as American cockroach, *Periplaneta Americana* (Blattodea: Blattidae), German cockroach, *Blattella germanica* (Blattodea: Ectobiidae), brown-banded cockroach, *Supella logipalpa* (Blattodea: Ectobiidae) and Oriental cockroach, *Blatta orientalis* (Blattodea: Blattidae) are found frequently in Iran (2). A number of faunistic studies of cockroaches in the human dwellings of Iran showed that *B. germanica* as the most frequent species followed by *P. americana* (3, 4). American and German cockroaches, respectively, were introduced as the prevalent species in human residential habitats (5). The German cockroach, which is approximately 15mm (0.59inch) long and the American cockroach, approximately 30mm (1.2inches) long (2, 6).

Cockroaches are able to transfer fungi, bacteria, viruses, parasites and other medically significant pathogenic agents on their body surfaces and in their feces in infectious regions, such as domestic habitats, hospitals, and industrial areas. From these insects collected from such environments have been isolated important pathogenic microorganisms (7–10). Plentiful pathogenic agents including 2 species of protozoans, 15 species of molds and fungi, 32 species of bacteria (such as, *Shigella* and *Salmonella*), 1 virus and 7 helminths which are damaging to humans being detected in the feces, in gut or on cuticle cockroaches (11–13).

Some fungi have the capacity to distribute via cockroaches (6, 14). It makes them ideal carriers for transferring a number of medically important fungi (15, 16). *Candida* spp., *Aspergillus* spp., *Penicillium* spp. and other species of fungi have been isolated from cockroaches recovered in several healthcare sectors of the hospitals (2, 6, 15–17). *Aspergillus* spp. and *Candida* spp. are the most prevalent fungi causing solemn healthcare-associated infections (18, 19). Aspergillosis is common in bone marrow transplant recipients and patients with lung disorders. In immunocom-promised patients, obstructive bronchial aspergillosis, allergic Aspergillus tracheobronchitis, and pulmonary aspergilloma are reported (20–23). *Candida* is known as an opportunistic pathogenic agent, due to it can innocuously colonize the human body (mouth, skin, genitourinary tract and gut). Candidiasis can cause symptoms when a weakened immune system or other factors allow it to grow unabated (24). To determine the possible role of American cockroaches in dissemination of medically important fungi, this study was carried out in residential areas of Iran. Fungi of medical importance were isolated from the American cockroaches' digestive system and identified.

## Materials and Methods

This research was carried out in Ahvaz (31°192 133 N 48°402 093 E) as a part of the central coordination Khuzestan (31.3273°N 48.6940°E), capital of Khuzestan, a southwestern province of Iran. In this descriptive study, 70 American cockroaches were sampled in Ahvaz, Iran in 2009–2010, from human dwelling localities using direct collection (hand catch), vacuum cleaner and sticky traps.

They were captured from kitchens, toilets or bathrooms of residential area. Each cockroach was placed in a single sterile test tube and transported to the laboratory for identification and processing for fungi examination. The cockroaches were immobilized by freezing at 0 °C for 10min. Each anesthetized cockroach was examined under the dissecting microscope, and the species were identified using standard taxonomic keys. After identification, 2ml of sterile normal saline (0.9%) was added to the test tube and the cockroaches were vigorously shaken for 2min. After external washing, the cockroaches were washed with 70% ethyl alcohol for 2min. Then the cockroaches transferred to sterilized tubes and allowed to dry. The cockroaches were then washed twice in sterile normal saline for 3min to remove traces of alcohol, and the gut was dissected out aseptically. The gut was then macerated under aseptic conditions in 2ml of sterile normal saline. The resulting macerate was cultured on Sabouraud's dextrose agar with 0.05% chloramphenicol and incubated at 30 °C for 3wk. The different yeast and filamentous colonies were distinguished by microscopic and macroscopic trials. Yeasts were diagnosed by germ tube test, the presence of chlamydoconidia on Corn meal plus Tween 80 agar and by API ID32C-32100 system.

## Results

A total of 70 American cockroaches, *Periplaneta americana* were sampled from 3 residential locations (30, 20 and 20 from Kyanpars, Amaniae and Golestan areas, respectively). About 51.4% cockroaches (36/70) carried one or more species of medically important molds in digestive system and 55.7% (39/70) had one or more species of medically important yeasts in digestive system. About 88.6% (62/70) cockroaches collected were contaminated with one or more fungi species (mold or yeast). Overall, 23 species/genera of fungi were isolated from these areas. The fungi isolated from cockroaches in these locations are shown in Table 1. In this investigation, *Candida* spp. (74.3%) was the most yeast isolated in the gut of American cockroaches and *Rhodotrula* spp. (48.7%), *Zygosaccharomyces* spp. (15.4%) and *Debaryomyces polymorphus* (2.6%) were the next. In addition, *Aspergillus* spp. (72.2%), *Penicillium* spp. (22.2%) and *Rhizopus* spp. (13.9%) were the most molds appeared in the gut of American cockroaches.

**Table 1.** Fungi isolated from the digestive system of *Periplaneta americana* captured in three residential areas, Ahvaz city, southeastern Iran

**Fungi isolated**	**Kyanpars area No. (%)**	**Amaniae area No. (%)**	**Golestan area No. (%)**	**Total No. (%)**
*Aspergillus flavus*	4(13.3)	0(0)	1(5)	5(7.1)
*Aspergillus niger*	10(33.3)	0(0)	1(5)	11(15.7)
*Aspergillus terreus*	2(6.7)	0(0)	0(0)	2(2.9)
*Aspergillus* sp.	4(13.3)	4(20)	0(0)	8(11.4)
*Rhizopus* sp.	2(6.7)	2(10)	1(5)	5(7.1)
*Penicillium* sp.	4(13.3)	4(20)	0(0)	8(11.4)
*Mucorales* sp.	0(0)	1(5)	0(0)	1(1.4)
*Alternaria* sp.	0(0)	1(5)	0(0)	1(1.4)
*Cladosporium* sp.	0(0)	1(1.7)	0(0)	1(0.5)
*Mycelia sterilia*	1(3.3)	2(10)	1(5)	4(5.7)
*Chrysosporium* sp.	0(0)	1(5)	0(0)	1(1.4)
*Candida albicans*	6(20)	0(0)	3(15)	9(12.9)
*Candida glabrata*	1(3.3)	0(0)	2(10)	3(4.3)
*Candida parapsilosis*	1(3.3)	0(0)	0(0)	1(1.4)
*Candida famata*	1(3.3)	1(5)	0(0)	2(2.9)
*Candida tropicalis*	0(0)	1(5)	0(0)	1(1.4)
*Candida guilliermondii*	1(3.3)	0(0)	0(0)	1(0.5)
*Candida krusie*	0(0)	1(5)	0(0)	1(1.4)
*Candida lipolytica*	0(0)	1(5)	0(0)	1(1.4)
*Candida* sp.	5(16.7)	1(5)	4(20)	10(14.3)
*Rhodotorula* sp.	5(16.7)	6(30)	8(40)	19(27.1)
*Zygosaccharomyces* sp.	3(10)	1(5)	2(10)	6(8.6)
*Debaryomyces polymorphus*	1(3.3)	0(0)	0(0)	1(1.4)
Yeasts	3(10)	5(25)	10(50)	18(25.7)

Other medically important mold, *Mycelia sterilia*, *Mucorales* spp., *Alternaria* spp., *Chrysosporium* spp. and *Cladosporium* spp. were rarely isolated from a few American cockroaches. Among 29 (41.4%) American cockroaches, nine species of *Candida* were identified by mycological tests. *Candida albicans* (31%), *C. glabrata* (10.3%) and *C. famata* (6.9%) were the greatest species isolated from cockroaches. Meanwhile, *C. parapsilosis*, *C guilliermondi*, *C. tropicalis*, *C. krusie*, *C. lipolytica* and *Candida* spp. were detected in the digestive canal in a few ones. Among 26 (37.1%) American cockroaches, four species of *Aspergillus* were identified. *Aspergillus niger* (42.3%) was the highest species isolated from American cockroaches. *Aspergillus flavus*, *A. terreus*, and *Aspergillus* spp. were detected in the digestive canal in a few American cockroaches.

## Discussion

The objective of this research was to isolate and identify the fungi found of gut of American cockroaches as a source of contamination. All cockroaches collected were identified as *P. americana*. Different pathogenic and nonpathogenic fungal agents were recovered from this cockroach in human environments. *Periplaneta americana* can carry pathogenic fungi in its internal organs. Therefore, *P. americana* is much more than a harassment and that it has important health hazards as a mechanical vector. Therefore, the abundance of American cockroaches’ population has to be reduced by various control methods, such as the proper management of garbage and organic waste disposal, sanitation and using safe insecticides. Clearly, presence of cockroaches in sensitive environments, hospitals and houses are more dangerous than other parts due to the special circumstances and the special people hospitalized and can affect to environmental, people and community health. Density of cockroaches in most parts of the hospital and residential dwellings as well as their feeding from secretions, human feces, and their ability to transmit a wide range of pathogenic agents, make it as ideal vector to transmit most medically important microorganisms. Nowadays, cockroaches have access to an infection source, human food and the place for food production, their role in the transmission of the disease is undeniable. The propensity of American cockroaches to move freely and dwell sewers, restrooms and drains can support to make the problem worse. Ability of cockroaches in the transmission of pathogens is emphasized in many types of research in this regard. Infectious agents carried by cockroaches can infect human, animal and food resources in some conditions (1, 3, 4, 6–10, 25).

This study confirmed that these insects in residential areas were contaminated with fungi of medical importance. A total of 12 yeast and 11 filamentous species of fungi were isolated from American cockroaches. In this study, a high percentage of the cockroach specimens (88.6%) from the houses were found to carry known fungal pathogens including *Penicillium* spp., *Candida* spp. and *Aspergillus* spp. Thus, the isolation of medically important fungi suggests a serious risk concern for patients. Although the direct involvement of American cockroaches in transmission of infectious agents is difficult to demonstrate. Other several studies have also isolated, from cockroaches from residential areas and hospitals, medically important fungi (14 –17, 25 –27). In the present study, the main fungi isolated were species of *Candida* spp. (41.4%), *Aspergillus* spp. (37.1%), *Rhodotrula* spp. (27.1%) and *Penicillium* spp. (11.4%). The findings from this study about medically important fungi isolated from cockroaches are agreed with the results of some workers. In a study in Thailand, *Penicillium* spp. and *Aspergillus* spp. appeared frequently on integument of 16 (35.6%) and 11 (24.4%) cockroaches, respectively (17). In another study in Brazil, *Candida* sp. (38.6%), *Aspergillus* sp. (30.7%) and *Penicillium* sp. (8.9%) were the most common fungi recovered on cockroaches (14). In addition, in Sari (Iran), *Candida* spp, *Aspergillus* spp., and *Rhodotrula* spp. were the most fungi appeared on cuticle of cockroaches (25). In a survey in Kashan (Iran), the prevalence of fungal agents in cockroaches was 41.1% and 22.8%, respectively. *Candida* spp. (39.5%), *Aspergillus* spp. (37, 2%) and *Penicillium* spp. (5.4%) had maximum prevalence among fungi observed (27). In India, *Candida* spp. and *Aspergillus* spp. were the most frequent fungi of medically important genera from cockroaches from a hospital and a residential area (29).

The finding of the present study also showed, *C. albicans* (31%), *C. glabrata* (10.3%) and *C. famata* (6.9%) were the greatest species isolated from cockroaches. *Candida glabrata* (42.2%) was the highest species isolated from cockroaches. The second highest was *C. magnoliae* which 17.8% of cockroaches contaminated (14). Yeast identification of *Candida* species showed a higher percentage of *C. glabrata* (15.4%), *C. parapsilosis* (15.4%) and *C. pseudotropicalis* (15.4%), than *C. albicans* (2.6%) isolated from cockroaches (17). *Candida glabrata* (52.8%) and *C. albicans* (38.8%) were the highest species isolated from cockroaches. In the present study, four species of *Aspergillus* were identified. *Aspergillus niger* (42.3%) and *A. flavus* (19.2%) were the highest species isolated from American cockroaches. *Aspergillus niger* (50%) was the most species isolated from cockroaches. Moreover, *A. flavus* and *A. fumigatus* were the most frequently recovered species from cockroaches (14). *Aspergillus niger* was significantly more frequent in the residential area and the hospital (28). In a study in the hospital environments in Ahvaz City, 28 fungal species were isolated from adult housefly. The main fungi isolated were *Aspergillus* spp. (67.4%), *Penicillium* sp. (11.6%), *Mucorales* sp. (11%), *Candida* spp. (10.5%), and *Rhodotorula* sp. (8.4%) (29).

We have displayed that American cockroaches transport great number of species of medically significant fungi in their digestive system, incriminated as significant agents in nosocomial infections. Hospital-acquired fungal infections are considered consequential causes of morbidity in immunocompromised individuals especially in those remained in hospital for a long period (30). *Aspergillus* sp., an important medical species isolated in our study, has been reported in hospital-acquired infections. *Aspergillus flavus*, the species isolated in this study, had been isolated in bone marrow transplant recipients (31). Moreover, *A. niger* and *A. flavus* have been reported from patients with invasive disease (32–35). Furthermore, other mold species as *Penicillium* spp., *Alternaria* spp., *Cladosporium* spp., *Mucorales* spp. and *Chrysosporium* spp. have lately appeared as significant pathogenic microorganisms inappropriately unable persons (25).

Among *Candida* species, *C. albicans* was the commonest species of this genus. *Candida* is noticed as an opportunistic pathogenic microorganism. *Candida albicans* responsible for the majority of hospital-acquired infections. *Candida albicans* is the most prevalent fungi in healthy people, moreover the most prevalent fungal pathogenic agent causing deadly infections (especially in immunocom-promised subjects). *Candida tropicalis*, *C. glabrata*, *C. guilliermondii*, and *C. parapsilosis* have lately emerged as significant infectious agents inappropriately unable people (36). *Rhodotorula* species have been reported as nosocomial meningitis and endophthalmitis, particularly in HIV infected persons (25).

Cockroaches living near human environments were significant vectors of etiological agents and all groups of possible pathogens such as protozoans, bacteria, helminths, and viruses. Various bacteria universally associated with these insects are recognized to inure diarrhea, dysentery, and food intoxication in humans. Cockroaches have been associated with an outbreak of dysentery. Different species of bacteria of public health significance have been isolated from *Periplaneta americana*, such as *Staphylococcus aureus*, *Streptococcus* spp., *Enterobacteriaceae*, *Pseudomonas aeruginosa*, and so on. Cockroaches captured in hospitals and houses have been found to harbor multi-drug resistant bacteria (9).

Therefore, the high contamination of cockroaches to the agents of infectious diseases can transmit fungal infections in public places such as hospitals and the home. Hospitals and homes are the focus of infection and a good place to cockroaches can transfer infectious agents. Therefore, the occurrence of infected cockroaches in the sensitive environment of hospital and home, that are places for treatment of patients and living of people, is very dangerous and threatening public health. Therefore, control of these insects is essential to achieve the essential goals of hospitals and to create a safe environment in homes.

## Conclusion

We revealed the presence of pathogenic filamentous fungi and yeasts in the gut of *Periplaneta americana* collected from the houses in the city of Ahvaz. Therefore, American cockroaches are a potential vector of pathogenic fungal microorganisms in residential environments. The control of *P. americana* in residential dwellings is essential in order to control the fungal infections in people.
